# Multi-omics integration reveals the impact of mediterranean diet on hepatic metabolism and gut microbiota in mice with metabolic dysfunction-associated steatotic liver disease

**DOI:** 10.3389/fnut.2025.1644014

**Published:** 2025-08-12

**Authors:** Zixuan Wang, Ge Chen, Xutong Sun, Jia Xiao, Lingling Kong, Shunshun Jiang, Tingting Xu, Meijiao Wang, Hong Zhao

**Affiliations:** ^1^Qingdao Medical College of Qingdao University, Qingdao, China; ^2^Department of Gastroenterology, Qingdao Central Hospital, University of Health and Rehabilitation Sciences, Qingdao, China

**Keywords:** metabolic dysfunction-associated steatotic liver disease, mediterranean diet, dietary intervention, hepatic metabolism, gut microbiota, omics analysis

## Abstract

**Objective:**

To investigate the effects of the Mediterranean diet (MD) on hepatic metabolism and gut microbiota in mice with metabolic dysfunction-associated steatotic liver disease (MASLD).

**Methods:**

C57BL/6 mice were fed a high-fat diet for 12 weeks to induce MASLD, with normal chow (NC)-fed mice as controls. Post-modeling, MASLD mice were randomized into three groups: HF (continued high-fat diet), HF-NC (switched to normal chow), and HF-MD (switched to MD). After 18-week interventions, body/liver weights, serum liver enzymes (ALT, AST), hepatic glycolipid markers (glucose, TC, TG, IBIL, DBIL), inflammatory cytokines (IL-6, TNF-α; ELISA), and histopathology (H&E and Oil Red O staining) were analyzed. Gut microbiota (metagenomic sequencing) and short-chain fatty acids (SCFAs; targeted metabolomics) were profiled.

**Results:**

High-fat diet induced MASLD features including obesity, increased abdominal fat mass, hepatic steatosis with lipid droplets, and inflammation. Both HF-NC and HF-MD groups exhibited reduced body weight, liver index, hepatic cytokines, serum enzymes, and improved glucolipid profiles vs. HF group (*p* < 0.05), with histopathology confirming attenuated steatosis. HF-MD outperformed HF-NC in lowering ALT, AST, IL-6, and TNF-α (*p* < 0.05). MASLD mice showed gut dysbiosis characterized by decreased diversity, elevated *Alistipes, Helicobacter, Mucispirillum*, and *Chlamydia*, reduced SCFAs, and increased branched-chain fatty acids (BCFAs) (*p* < 0.05). Both dietary interventions partially ameliorated gut dysbiosis in MASLD mice, with the HF-MD group uniquely enriching beneficial taxa including *Prevotella, Muribaculum, Duncaniella*, and *Barnesiella*.

**Conclusion:**

MD alleviates MASLD progression by synergistically improving hepatic metabolic homeostasis and gut microbiota composition, demonstrating superior efficacy over NC in mitigating inflammation, enriching beneficial microbes, and regulating microbial metabolism. These findings highlight MD's potential as a targeted dietary intervention for MASLD.

## 1 Introduction

Metabolic dysfunction-associated steatotic liver disease (MASLD) is characterized by the buildup of surplus triglycerides in the liver, occurring alongside at least one cardiometabolic risk factor (1). This definition was officially put forward in the “Multisociety Delphi Consensus Statement” in 2023, which was discussed and reached by the American Association for the Study of Liver Diseases (AASLD), the European Association for the Study of the Liver (EASL), and the Asociacion Latinoamericana para el Estudio del Hígado (ALEH). MASLD replaces the terms “non-alcoholic fatty liver disease (NAFLD)” and “metabolic-associated fatty liver disease (MAFLD)”. Its disease spectrum covers metabolic dysfunction-associated steatotic liver (MASL), metabolic dysfunction-associated steatohepatitis (MASH), as well as related fibrosis, cirrhosis, and hepatocellular carcinoma (HCC) (2). Findings from existing cohort studies support the applicability of NAFLD-related discoveries to MASLD patients (3).

MASLD, recognized as one of the most challenging chronic liver diseases today, is rapidly becoming a global concern. Epidemiological data indicate that 38% of all adults and between 7% and 14% of children and teenagers are impacted by MASLD, and its prevalence is continuously on the rise (4). MASLD is not merely an independent disease entity within the realm of liver diseases but also forms a complex pathological network with metabolic syndrome (MS) and type 2 diabetes mellitus (T2DM). These three conditions are interconnected via mechanisms such as metabolic aberrations and inflammatory responses, synergistically augmenting the risk of cardiovascular, cerebrovascular, and renal vascular sclerotic lesions, as well as intra-and extra-hepatic malignancies (5). The elucidation of its pathogenesis remains full of mysteries. The current mainstream “multiple parallel hit” theory (6) postulates that factors such as insulin resistance, oxidative stress damage, lipid metabolism disorders, pro-inflammatory cytokine cascades, gut microbiota dysbiosis, and genetic predisposition constitute a multi-dimensional hit system, jointly propelling the onset and progression of MASLD. In recent years, the gut-liver axis (GLA) theory has drawn substantial attention as an explanation for the pathogenesis of MASLD (7). As a bidirectional conduit linking the liver and the gut, the GLA is essential for preserving metabolic balance by ensuring the integrity of the intestinal mucosal barrier, a dynamically balanced gut microbiota and its metabolites, and a precisely regulated immune interaction network (8). Studies have revealed that MASLD patients at different disease stages display characteristic gut microbiota profiles (9). Nevertheless, the lack of consensus on microbiota dysbiosis signatures and their pathophysiological consequences (10) underscores the imperative to delineate gut microbial mechanisms driving MASLD pathogenesis.

No pharmacotherapy has been formally approved for MASLD, positioning lifestyle modification as the cornerstone therapeutic strategy throughout disease progression. The joint clinical guidelines from European associations (EASL-EASD-EASO) strongly recommend MD as the primary nutritional intervention for MASLD patients (1). While clinical trials consistently demonstrate MD's capacity to improve hepatic transaminases and attenuate steatosis in MASLD (11), heterogeneity persists regarding its impacts on gut microbial composition and metabolic functionality (12). Furthermore, limited preclinical evidence exists, potentially attributable to methodological constraints in capturing the multidimensional diet-microbiota-host interactome.

Emerging multi-omics technologies hold transformative potential for overcoming current research limitations. Metagenomic sequencing enables high-throughput profiling of gut microbiota composition, while targeted metabolomics precisely quantifies microbial metabolites. Together, they facilitate exploration of diet-microbiota-metabolism-host interactions. However, integrated multi-omics studies on the mechanisms of metabolic remodeling in MASLD under dietary intervention remain scarce, particularly regarding the dynamic associations between microbial functional shifts and improvements in host metabolic phenotypes. This study employed a high-fat diet-induced MASLD mouse model to investigate disease progression and recovery. Using integrated metagenomics and targeted metabolomics, we assessed gut microbiota composition and associated metabolites. We comprehensively examined the effects of Mediterranean Diet (MD) intervention on hepatic lipid metabolism, inflammatory responses, and gut microbiota functionality. Our findings elucidate the ameliorative effects of MD on MASLD, paving the way toward the clinical translation of precision nutrition for MASLD patients.

## 2 Materials and methods

### 2.1 Experimental model establishment

**Figure 1:**
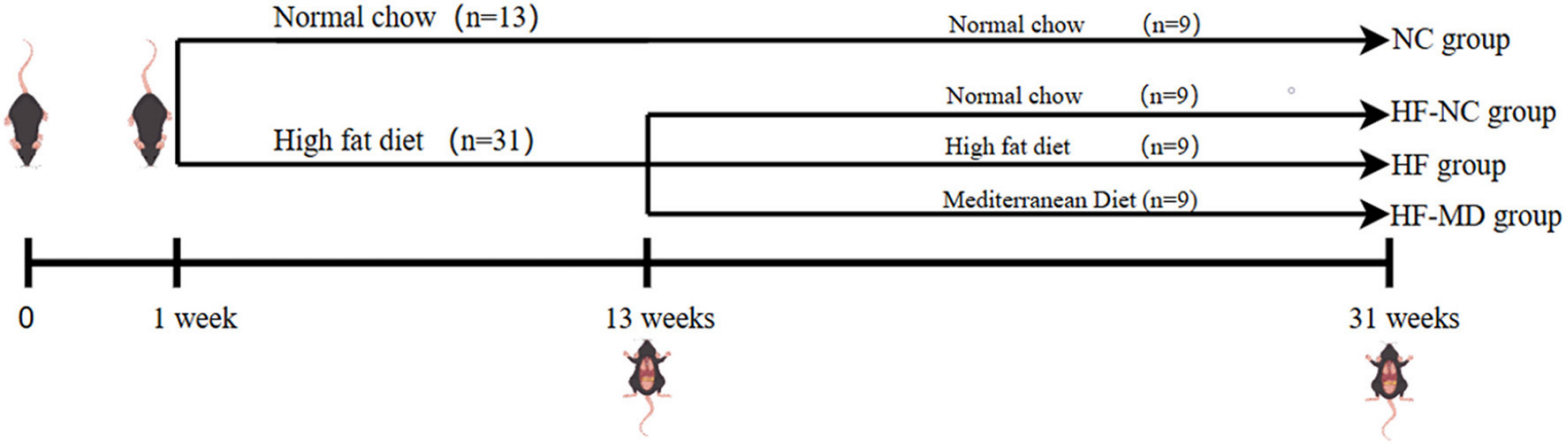
Diagrammatic representation of experimental design.

A total of forty-four male C57BL/6 SPF mice, aged 4 weeks, were acclimatized for 1 week under controlled conditions (12 h light/dark cycle, 22 ± 3°C) and fed normal chow (20% protein, 10% fat, 70% carbohydrates). Figure 1 outlines the workflow of the experimental protocol. Animals were then randomized into a control group (*n* = 13, normal chow) and a model group (*n* = 31, high-fat diet: 20% protein, 60% fat, 20% carbohydrates) for 12-week MASLD induction. Successful modeling was confirmed via serum biochemistry and histopathology in 4 mice/group at week 12. Remaining MASLD mice were reallocated into three cohorts (*n* = 9/group): HF (continued high-fat diet), HF-NC (switched to normal chow), and HF-MD (switched to MD) (MD: 15% protein, 35% fat, 50% carbohydrates). Control mice continued to be fed a normal chow named NC group (*n* = 9). Body weight and food intake were monitored weekly during the 18-week intervention, followed by terminal tissue collection. All procedures complied with ethical guidelines (Approval No. KY202405701, Qingdao Central Hospital).

### 2.2 Specimen collection

Following a 12-h overnight fast with *ad libitum* water access, mice were anesthetized via intraperitoneal administration of 5% chloral hydrate (0.1 ml/10 g body weight) and blood samples were collected via retro-orbital puncture into EDTA-coated tubes, followed by euthanasia via cervical dislocation. Hepatic and epididymal adipose tissues were immediately excised for morphological imaging and gravimetric analysis (liver index = fresh liver weight/body weight). Liver specimens were processed into paraffin-embedded sections, cryosections, and snap-frozen aliquots (−80°C), while adipose tissues were preserved as cryosections and frozen samples. Fresh fecal samples (≥100 mg) were stabilized in 0.1% HCl-containing buffer prior to freezing. Whole blood in the EP tube was allowed to stand undisturbed for 2 h at room temperature. Subsequently, serum was separated by centrifugation (4,000 r/min, 15 min) and stored at −20°C for subsequent experiments.

### 2.3 Serum biochemical profiling

Serum concentrations of alanine aminotransferase (ALT), aspartate aminotransferase (AST), alkaline phosphatase (ALP), gamma-glutamyl transferase (γ-GT), fasting glucose (Glu), total cholesterol (TC), triglycerides (TG), low-density lipoprotein cholesterol (LDL-C), high-density lipoprotein cholesterol (HDL-C), total protein (TP), total bile acid (TBA), total bilirubin (TBIL), unconjugated bilirubin (IBIL), and conjugated bilirubin (DBIL) were quantitatively analyzed using commercial assay kits (Nanjing Jiancheng Bioengineering Institute, China) with an XR220 Plus automatic biochemical analyzer (Xinrui, China). All biochemical determinations were performed in strict accordance with the manufacturer's standardized protocols, including calibration procedures and quality control measures as specified in the technical documentation.

### 2.4 Quantification of hepatic inflammatory mediators

Quantification of interleukin-6 (IL-6) and tumor necrosis factor-alpha (TNF-α) in murine hepatic tissues was performed using commercially available ELISA kits (Medical Discovery Leader, China), in accordance with the manufacturer's standardized protocols.

### 2.5 Comprehensive histopathological assessment

The liver tissue samples underwent histopathological examination, with paraffin-embedded sections stained with hematoxylin and eosin (H&E) to evaluate cellular structure. Meanwhile, frozen liver sections were treated with Oil Red O stain to detect lipid accumulation. Additionally, morphological characterization of adipose tissue was conducted through H&E staining of frozen sections to evaluate structural alterations.

### 2.6 Fecal DNA extraction and metagenomic sequencing

Genomic DNA was isolated from the samples using the CretMagTM Power Soil DNA Kit (Cretaceous, China). After extraction, DNA concentration was quantified with TBS-380, DNA purity was assessed with a NanoDrop200 (Thermo Fisher Scientific, USA), and DNA integrity was confirmed via 1% agarose gel electrophoresis. Fragment optimization was performed using magnetic bead-based size selection, targeting 200–400 bp fragments. Subsequent library preparation included end repair, A-tailing, and adapter ligation. PCR amplification is performed, the amplified product is recovered by product purification using magnetic beads, and the PCR product is cyclized to give a final library for pre-machine concentration testing. The final libraries were subjected to pre-sequencing (DNBSEQ, China).

### 2.7 Microbial analysis

Fastq software (13) processed raw Illumina sequencing data for quality assessment. Sequencing reads were assembled with MEGAHIT (14) (https://github.com/voutcn/megahit, version 1.1.2). Binning analysis was performed using vamb software and the results were screened against the following criteria: genome integrity >50% and contamination rate < 10%. Unigenes were aligned with sequences of bacteria, fungi, archaea and viruses extracted from NCBI's NR (Version: 2021.11) database using DIAMOND (15) software (blastp, evalue ≤ 1e−5) (16). Alpha diversity was measured to assess species diversity per sample. β-diversity was estimated using Bray-Curtis distance based on principal coordinate analysis (PCoA), non-metric multidimensional scale analysis (NMDS), and principal component analysis (PCA) as appropriate. Statistical comparisons of bacterial abundance and diversity were conducted using the Wilcoxon rank-sum test and Welch's *t*-test. Heatmaps visualizing phylum- and genus-level taxonomic profiles were generated based on non-parametric Wilcoxon test results (*p* < 0.05). Taxa with significant abundance differences were assessed using LEfSe (LDA effect size) analysis. Inter-group differential analysis of gut microbiota was performed using STAMP software. The Kyoto Encyclopedia of Genes and Genomes (KEGG) pathway analysis (KOBAS) with FDR correction (Benjamini-Hochberg, *p* < 0.05) identified significantly enriched pathways in differentially expressed genes.

### 2.8 Quantitative analysis of gut-derived SCFAs via targeted metabolomics

Fecal samples were homogenized in 2 ml EP tubes with 1 ml of ultrapure water by vortexing (10 s) and mechanical disruption using steel beads in a tissue homogenizer (40 Hz, 4 min). Subsequent steps included ice-water bath ultrasonication (20 kHz, 5 min, repeated 3 × ) and centrifugation (5,000 rpm, 20 min, 4°C). A total of 0.8 ml of supernatant was transferred to a new 2 ml EP tube, mixed with 0.1 ml of 50% (v/v) sulfuric acid and 0.8 ml of extraction solution containing 2-methylvaleric acid (internal standard, 25 mg/L) in methyl tert-butyl ether. The mixture was vortexed (10 s), shaken (10 min), sonicated (10 min, ice-cooled), then spun (10,000 rpm, 15 min, 4°C). After static incubation (−20°C, 30 min), the supernatant was transferred to injection vials. SCFAs were quantified using a Shimadzu GC2030-QP2020 NX GC-MS system equipped with an Agilent HP-FFAP capillary column (30 m × 250 μm × 0.25 μm). Electron ionization (70 eV) and selected ion monitoring (SIM) were employed for detection. Quantitation was performed via internal standard calibration (linear range: 0.02–100 mg/L, R^2^ > 0.99). Quality controls included blank samples, spiked recoveries (80%−120%), and technical replicates (RSD ≤ 20%).

### 2.9 Statistical analyses

Statistical analyses were done with GraphPad Prism 9 (GraphPad Software, San Diego, CA). Continuous data are shown as mean ± standard deviation (SD). Intergroup comparisons were conducted via unpaired Student's *t*-tests, while multigroup analyses utilized one-way ANOVA with *post hoc* Tukey's tests.

## 3 Results

### 3.1 Establishment of a mice MASLD model via 12-week high-fat diet feeding

**Figure 2:**
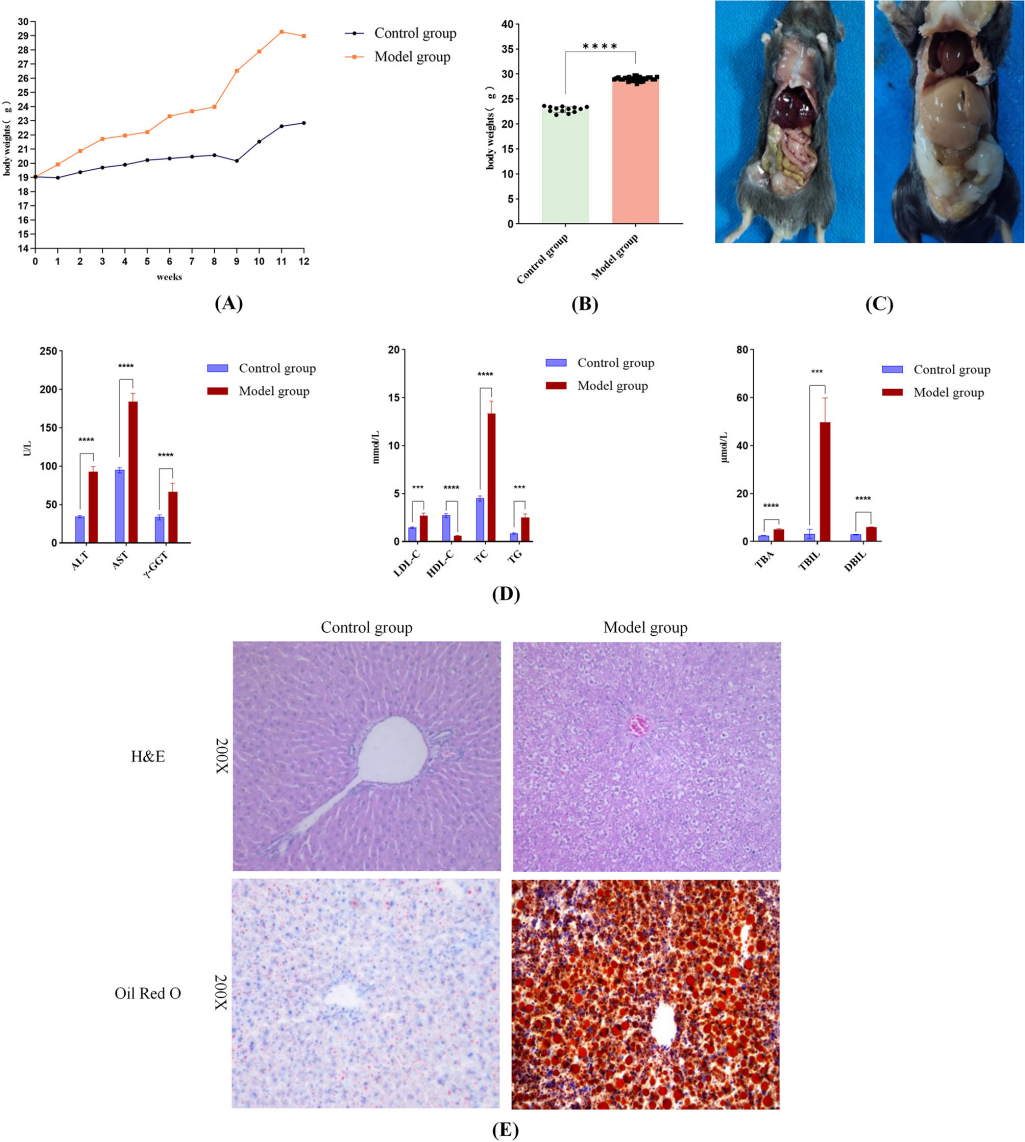
Successful induction of MASLD in mice following 12-Week high-fat diet feeding. **(A)** Body weight changes in the model and control groups during the 12-week modeling period. **(B)** At week 12, the body weight of the mice in the model group was notably greater than that of the control group (*p* < 0.05). **(C)** Gross anatomical photographs of mice in the control and model groups. **(D)** Serum levels of ALT, AST, γ-GT, LDL-C, TC, TG, TBA, TBIL, DBIL were considerably higher in the model group in comparison to the control group, along with a marked reduction in HDL-C (*p* < 0.05 for all comparisons). **(E)** Histopathological features of liver tissue: Model group showing lipid droplets and ballooning degeneration. Significance thresholds were defined as follows: ***p < 0.001, ****p < 0.0001 vs. control group. (Only significant differences are marked).

Following 12 weeks of a high-fat diet, the model group showed a notable increase in body mass in comparison to the control group (Figure 2A). Gross anatomical observations (Figure 2C) revealed that mice in the model group displayed enlarged body size, excessive intra-abdominal adiposity, and hepatomegaly characterized by pale discoloration, greasy appearance, soft consistency, mottled surface texture, and rounded hepatic margins. Conversely, mice in the control group maintained normal body proportions, minimal abdominal fat deposition, and livers with physiological dimensions, bright red coloration, firm texture, smooth glistening surfaces, and well-defined margins.

Serological analysis (Figure 2D) demonstrated that the model group exhibited significantly elevated levels of hepatic injury markers (ALT, AST, γ-GT), lipid metabolism parameters (LDL-C, TC, TG, TBA), and bilirubin profiles (TBIL, DBIL) compared to the control group, alongside a pronounced decrease in HDL-C (*p* < 0.05 for all comparisons). These biochemical alterations collectively indicated hepatic dysfunction and steatosis. Histopathological evaluation further corroborated these findings: HE staining revealed hepatic steatosis, hepatocyte ballooning, and inflammatory cell infiltration in portal areas, while Oil Red O staining highlighted extensive lipid droplet accumulation and vacuolar degeneration in the model group (Figure 2E). Thus, histopathology further confirms that the high-fat diet caused pathological changes in mouse livers, including lipid accumulation and hepatocyte injury. Integrated analysis confirmed the successful establishment of a MASLD mouse model in the model group following 12 weeks of high-fat diet intervention, recapitulating hallmark pathological features including dyslipidemia, ectopic lipid deposition, and progressive liver injury.

### 3.2 18-week dietary intervention in MASLD mice

#### 3.2.1 Effects of MD on growth parameters in MASLD mice

**Figure 3:**
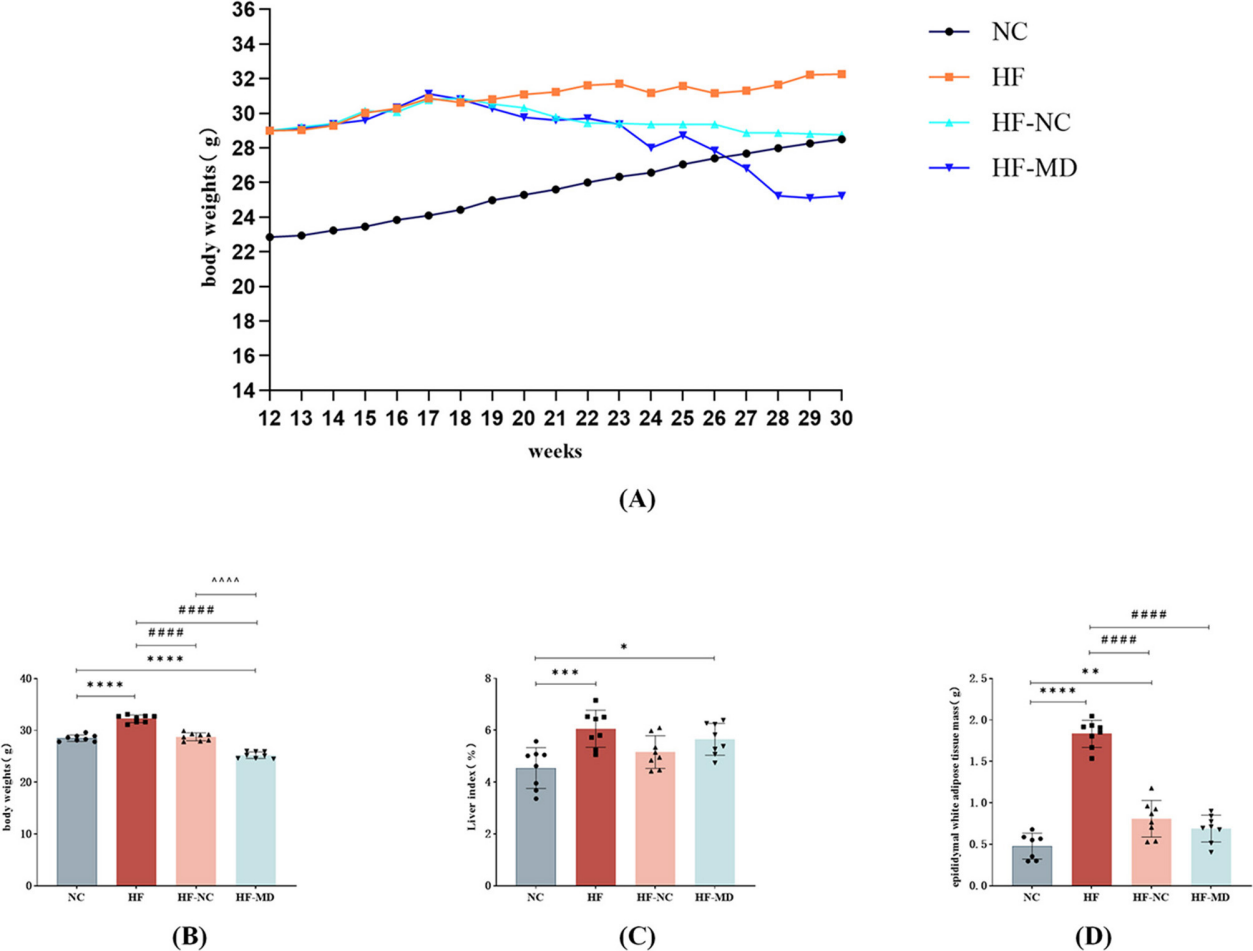
Effects of dietary interventions on growth parameters in MASLD mice: **(A)** body weight trajectories; **(B)** inter-group body weight differences at intervention endpoint; **(C)** liver index comparisons across groups at intervention endpoint; **(D)** epididymal white adipose tissue (eWAT) mass variations among groups at intervention endpoint. Significance thresholds were defined as follows: *p < 0.05, **p < 0.01, ***p < 0.001, ****p < 0.0001 vs. NC group; ^####^*p* < 0.0001 vs. HF group; ^ΛΛΛΛ^*p* < 0.0001 vs. HF-NC group (Only significant differences are marked).

Following 18 weeks of dietary intervention, body weight trajectories diverged significantly: the NC group exhibited steady weight gain, whereas the HF group demonstrated a markedly steeper weight gain trend. Both NC and MD interventions attenuated body weight elevation in MASLD mice, with the HF-MD group showing a more pronounced reduction compared to the HF-NC group (Figures 3A, B).

Compared with the NC group, the HF group exhibited a marked increase in liver index (liver-to-body weight ratio; *p* < 0.01). Although the HF-NC and HF-MD groups exhibited slight decreases in liver index, these reductions were not statistically significant (*p* > 0.05), and values remained higher than those in the NC group (Figure 3C).

Epididymal white adipose tissue (eWAT) mass analysis revealed significantly greater fat deposition in the HF group compared to all other groups (*p* < 0.01). Both HF-NC and HF-MD interventions reduced eWAT mass (Figure 3D).

#### 3.2.2 Effects of MD on fasting glucose and lipid metabolism in MASLD mice

**Figure 4:**
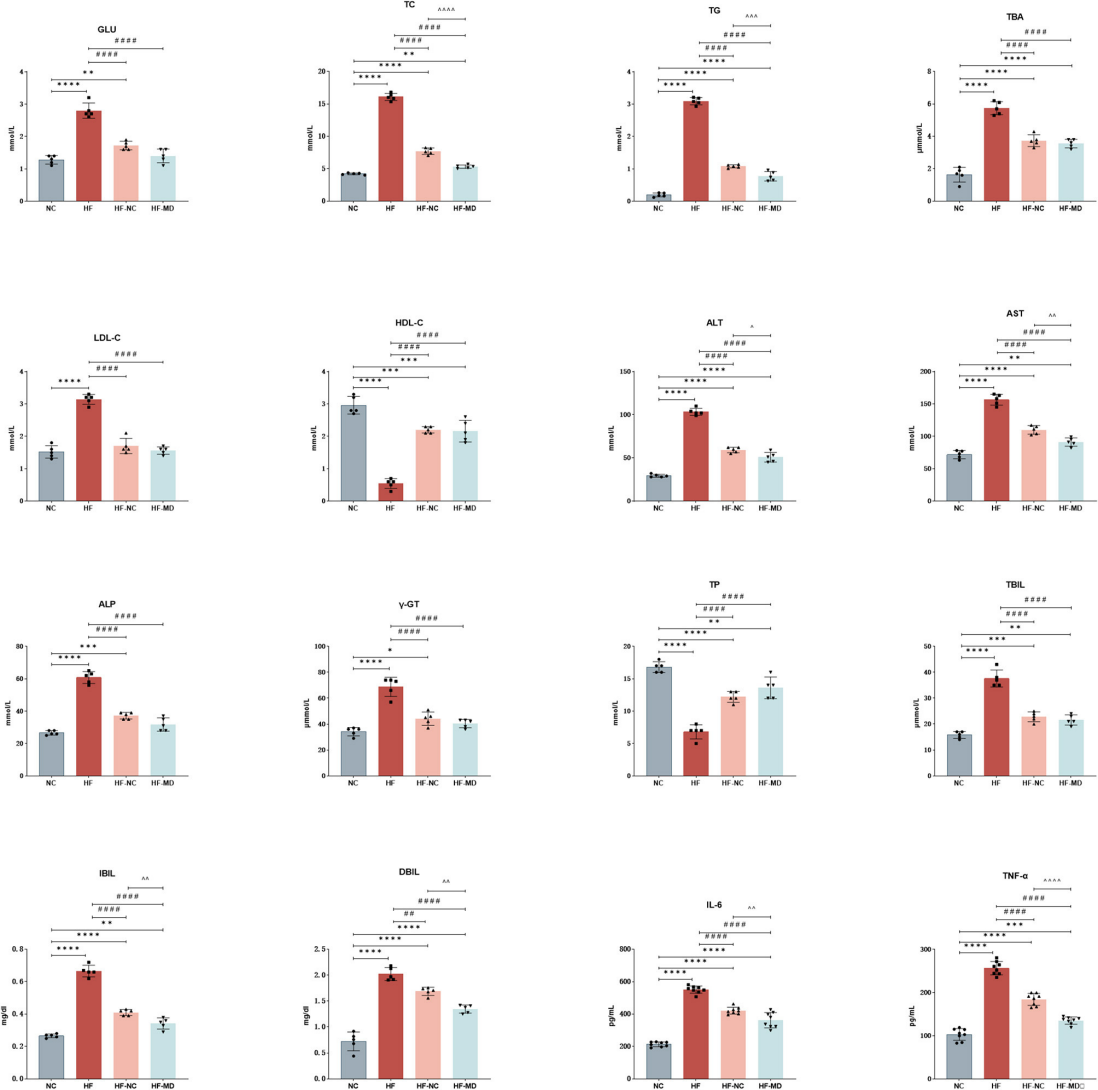
Improvement of hepatic enzymes and synthetic function in MASLD mice after 18-week intervention with normal chow and MD dietary regimens: both HF-NC and HF-MD groups exhibited significant improvements in hepatic glucose-lipid metabolic parameters, serum hepatic enzymes, and pro-inflammatory cytokines compared to the HF group (*p* < 0.05). Notably, the HF-MD group demonstrated more pronounced reductions in serum ALT, AST, TC, TG, IBIL, DBIL, IL-6, and TNF-α levels relative to the HF-NC group (*p* < 0.05). Significance thresholds were defined as follows: *p < 0.05, **p < 0.01, ***p < 0.001, ****p < 0.0001 vs. NC group; ^##^*p* < 0.01, ^####^*p* < 0.0001 vs. HF group; ^ΛΛ^*p* < 0.01, ^Λ*ΛΛ*^*p* < 0.001, ^ΛΛΛΛ^*p* < 0.0001 vs. HF-NC group (Only significant differences are marked).

As illustrated in Figure 4, HF group exhibited significantly elevated Glu and serum lipid parameters compared to NC group, including TC, TBA, TG, and LDL-C, accompanied by reduced HDL-C (all *p* < 0.05). Both HF-NC and HF-MD groups demonstrated significant amelioration of these metabolic derangements in MASLD mice (Glu, TC, TBA, TG, LDL-C, and HDL-C, all *p* < 0.05). Notably, the HF-MD group achieved more pronounced reductions in TC and TG compared to HF-NC group (*p* < 0.01), though these parameters remained statistically higher than baseline NC levels.

#### 3.2.3 Effects of MD on hepatic enzymes and synthetic function in MASLD mice

As illustrated in Figure 4, serum levels of ALT, AST, ALP, and γ-GT were significantly elevated in the HF group compared to the NC group (*p* < 0.01). Both the HF-NC and HF-MD intervention groups exhibited marked reductions in these four hepatic enzymes relative to the HF group (*p* < 0.01), with the HF-MD group demonstrating more pronounced declines in ALT and AST levels compared to the HF-NC group.

Moreover, the HF group exhibited notably reduced levels of TP and increased serum levels of TBIL, IBIL, and DBIL in comparison to the NC group (*p* < 0.01). Dietary interventions restored TP levels in both the HF-NC and HF-MD groups (*p* < 0.01), while serum bilirubin and TBA were reduced in both intervention groups (*p* < 0.05). Notably, the HF-MD group exhibited significantly greater reductions in IBIL and DBIL compared to the HF-NC group.

These findings collectively suggest that both normal chow intervention and MD intervention partially restored hepatocyte integrity and functional capacity in MASLD mice.

#### 3.2.4 MD diet intervention ameliorates hepatic inflammatory response in MASLD mice

As demonstrated in the results (Figure 4), hepatic IL-6 and TNF-α levels in the HF group were significantly elevated compared to the NC group (*p* < 0.0001). Both the HF-NC and HF-MD intervention groups exhibited marked reductions in these pro-inflammatory cytokines relative to the HF group (*p* < 0.01). However, no statistically significant intergroup differences were observed between the HF-NC and HF-MD groups, with both remaining higher than the NC group. These findings suggest that both normal chow intervention and MD intervention partially mitigate hepatic inflammatory responses in MASLD mice.

#### 3.2.5 Histological effects of MD intervention on liver and adipose tissue architecture

**Figure 5:**
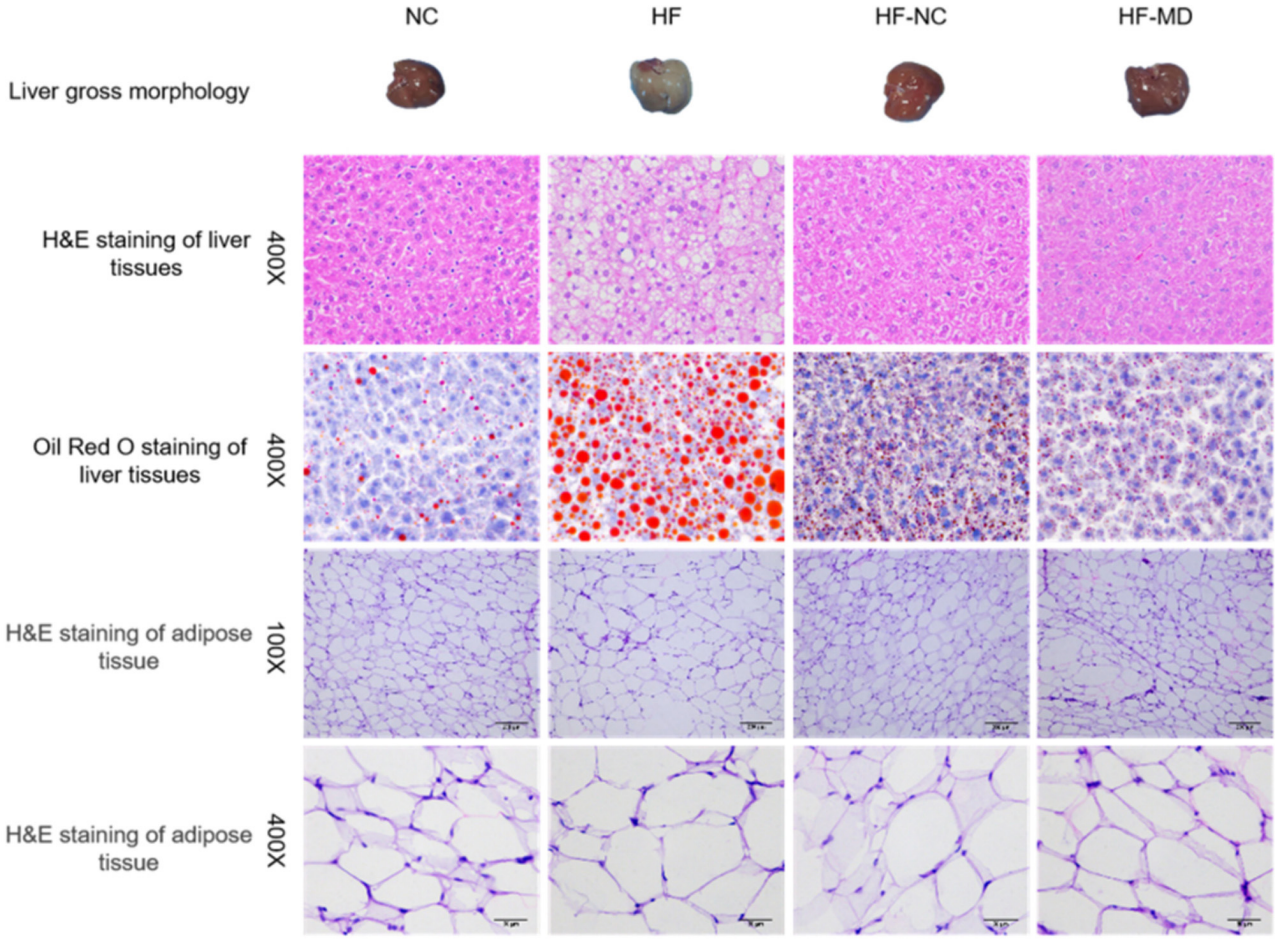
Gross morphology, H&E staining, and Oil Red O staining of liver tissues, along with H&E staining of adipose tissue, in mice from the NC, HF, HF-NC, and HF-MD groups.

Histological analysis revealed distinct liver pathology across groups (Figure 5). HE staining of liver tissues in the HF group revealed hepatocellular vacuolar degeneration, marked steatosis, and mild inflammatory cell infiltration, indicating aggravated overall pathological severity. After dietary interventions, both the HF-NC and HF-MD groups exhibited significant improvement in hepatic degeneration, with histopathological features approaching those in the NC group. Oil Red O staining demonstrated abundant lipid droplet accumulation and vacuolar degeneration in the HF group, whereas normal chow and MD interventions markedly reduced intrahepatic lipid deposition. For adipose tissue, HE staining indicated reduced adipocyte count, hypertrophic cell morphology, and cellular damage in the HF group, which showed amelioration following dietary interventions.

#### 3.2.6 Gut microbiota profiling in MASLD mice pre- and post-mediterranean diet intervention

The metagenomic sequencing analysis of fecal samples identified a total of 8,863 microbial species, comprising 222 archaea, 8,428 bacteria, 176 viruses, and 37 fungi. The results demonstrated that bacterial species dominated the gut microbiota composition, accounting for approximately 95% of the detected microbial population, thereby establishing their crucial functional significance in intestinal microbial ecosystems.

**Figure 6:**
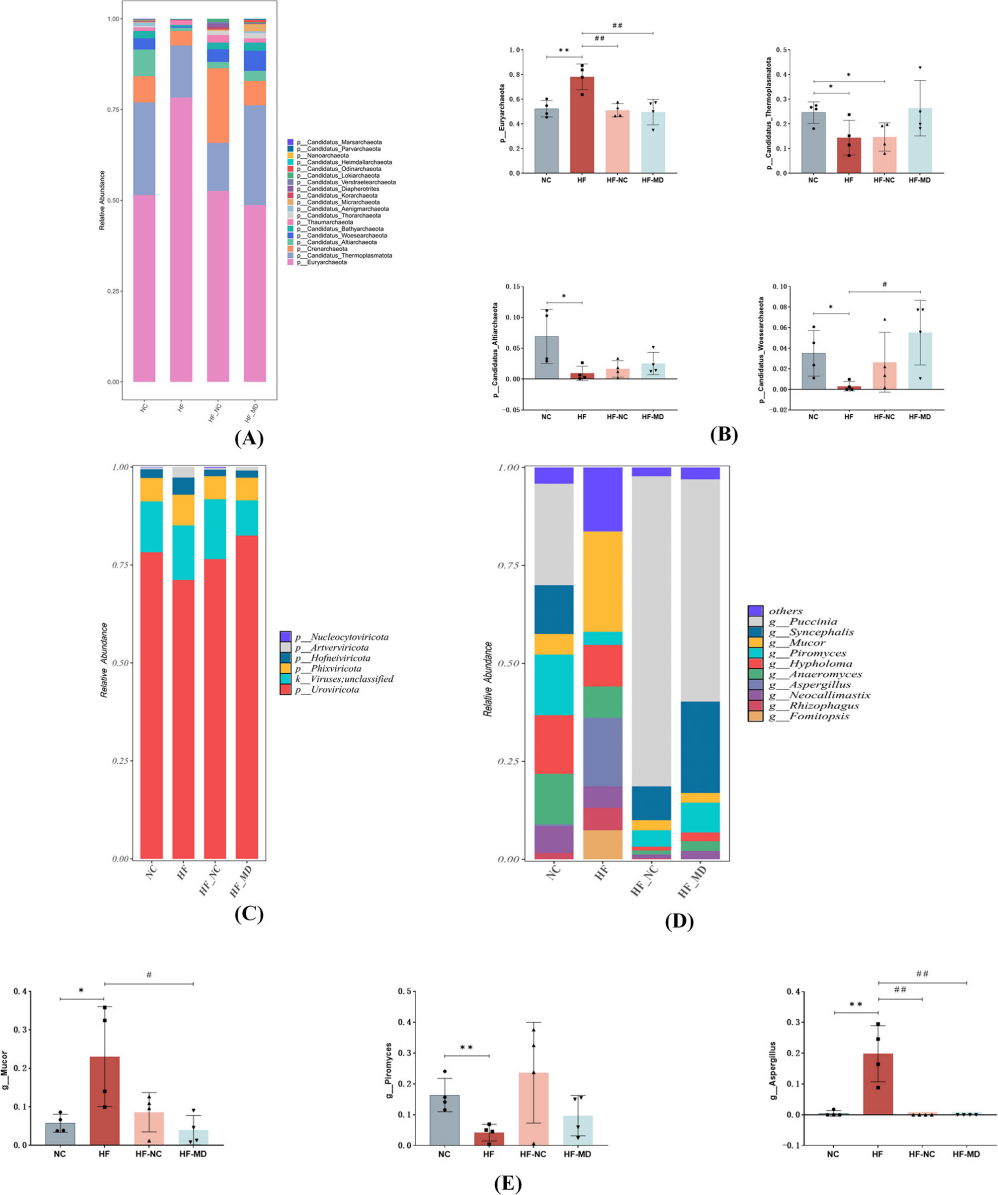
Alterations in the composition and structure of archaeal, viral, and fungal communities. **(A)** Relative abundance of archaea communities from mice in NC, HF, HF-NC and HF-MD group at the phylum. **(B)** Comparative analysis of differences in the relative abundance of four predominant archaeal taxa among the NC (normal control), HF (high-fat diet), HF-NC (high-fat diet + normal chow intervention), and HF-MD (high-fat diet + Mediterranean diet intervention) groups. **(C)** Relative abundance of viruses communities from mice in NC, HF, HF-NC and HF-MD group at the phylum. **(D)** Relative abundance of Fungal communities from mice in NC, HF, HF-NC and HF-MD group at the genus. **(E)** Comparative analysis of the relative abundance of three major differentially abundant fungal taxa among the NC, HF, HF-NC, and HF-MD groups. Significance thresholds were defined as follows: *p < 0.05, **p < 0.01 vs. NC group; ^#^*p* < 0.05, ^##^*p* < 0.01 vs. HF group (Only significant differences are marked).

Phylum-level relative abundance profiles of archaeal communities across experimental groups are shown in Figures 6A, B. At the phylum level, mice in the HF group exhibited a higher relative abundance of *Euryarchaeota* in comparison to the other groups, while showing a reduced relative abundance of *Candidatus Thermoplasmatota, Candidatus Altiarchaeota*, and *Candidatus Woesearchaeota* when contrasted with the NC group.

Phylum-level relative abundance profiles of viral communities across experimental groups are shown in Figure 6C. All groups exhibited low viral loads in the intestinal tract, with no significant compositional differences observed among the experimental groups at this taxonomic rank.

Fungal composition at the genus level is presented in Figures 6D, E. At the level of genus, the HF group exhibited significant restructuring of Fungal, characterized by a five-fold elevation in *Mucor* relative abundance compared to controls (*p* < 0.05), concurrent detection of *Aspergillus* (undetected in other groups), and a reduced relative abundance of *Piromyces* in comparison to the other groups. Dietary intervention induced partial mycobiota restoration, with the compositional profile approaching that of the NC group.

**Figure 7:**
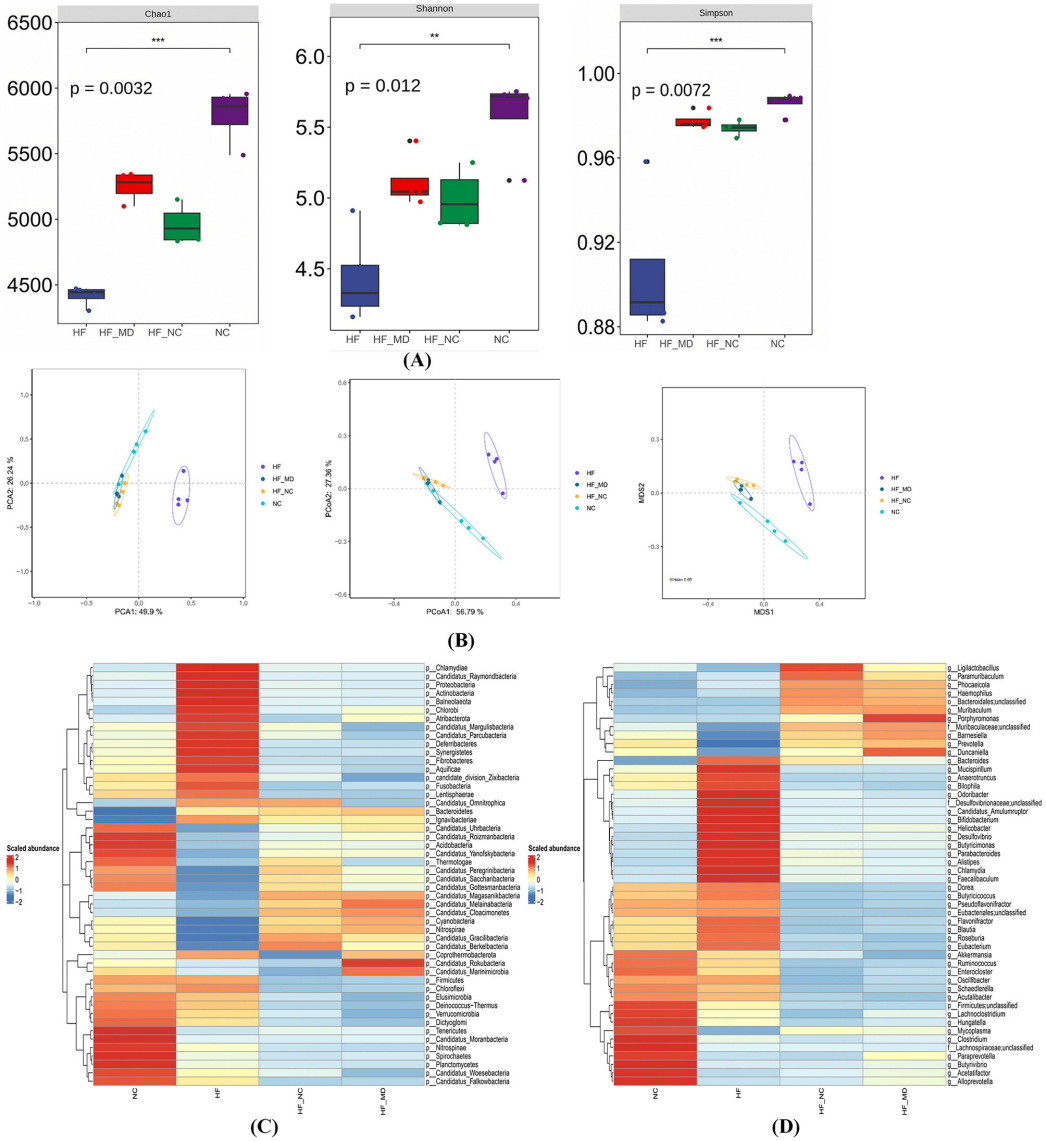
Gut microbial dysbiosis in MASLD mice, characterized by disrupted community structure and reduced diversity/richness, was partially restored by two dietary interventions. **(A)** α-diversity analysis of gut microbiota across four groups: Chao1 index, Shannon index, and Simpson index. **(B)** β-diversity analysis of gut microbiota: PCA analysis, PCoA analysis, and NMDS analysis based on Bray-Curtis dissimilarity. **(C)** Heatmap of gut bacterial abundance at the phylum level (top 50 most abundant taxa) in NC, HF, HF-NC, and HF-MD groups. **(D)** Heatmap of gut bacterial abundance at the genus level (top 50 most abundant taxa) across the four groups. Color gradients reflect Z-score normalized abundance values (scale: +2 to −2), with warm colors indicating higher relative abundance.

In this study, we conducted a thorough examination of bacteria, the main microbial domain found in the gut microbiota. Mice in the HF group displayed significantly lower Chao1, Shannon, and Simpson indices (*p* < 0.05) when compared to the NC group, suggesting a decline in both the richness of gut microbial species and the evenness of the community. Notably, both the HF-NC and HF-MD groups showed significant increases in these alpha-diversity indices relative to the HF group (*p* < 0.05) (Figure 7A).

Gut microbiota structural variations were assessed using PCA, Bray-Curtis distance-based PCoA, and NMDS. Multi-method analyses revealed complete segregation of the HF group's microbial communities from the other three groups (NC, HF-NC, HF-MD), while partial clustering overlap was observed among the NC, HF-NC, and HF-MD groups (Figure 7B).

Taxonomic stratification of gut bacteria at both phylum and genus ranks, expressed as relative abundance, is depicted in Figures 7C, D.

**Figure 8:**
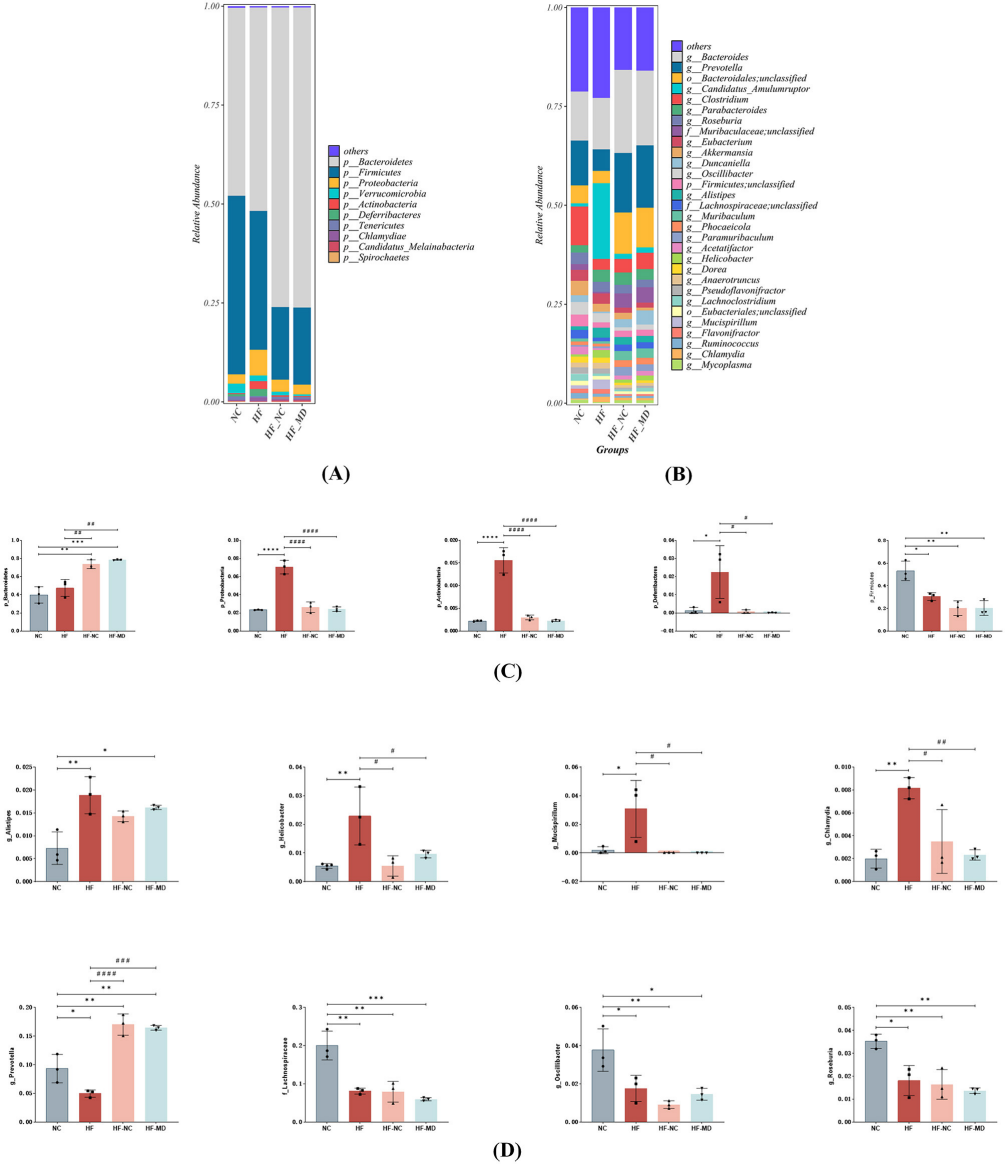
The MD intervention led to a partial restoration of the gut microbiome in mice with MASLD. The relative abundance of bacterial communities in mice from the NC, HF, HF-NC, and HF-MD groups is presented at both the phylum **(A)** and genus levels **(B)**. Additionally, a comparison of the relative abundance of specific gut bacteria among the mice in the NC, HF, HF-NC, and HF-MD groups is shown at the phylum **(C)** and genus levels **(D)**. Significance thresholds were defined as follows: *p < 0.05, **p < 0.01, ***p < 0.001, ****p < 0.0001 vs. NC group; ^#^*p* < 0.05, ^##^*p* < 0.01, ^###^*p* < 0.001, ^####^*p* < 0.0001 vs. HF group (Only significant differences are marked).

An examination of taxonomic variations at the phylum level (Figure 8A) showed that the composition of gut microbiota in all experimental groups was mainly made up of *Firmicutes, Bacteroidetes, Verrucomicrobia, Proteobacteria*, and *Actinobacteria*, with *Firmicutes* and *Bacteroidetes* together representing the most prevalent phyla. HF group mice exhibited significantly increased relative abundances of *Proteobacteria, Actinobacteria*, and *Deferribacteres* compared to the NC group (*p* < 0.05), whereas *Firmicutes* abundance was markedly reduced (*p* < 0.05). Both HF-NC and HF-MD groups demonstrated significant reductions in the relative abundances of these four bacterial phyla relative to the HF group. Notably, while Firmicutes levels in intervention groups diverged from NC group values, *Proteobacteria, Actinobacteria*, and *Deferribacteres* displayed ameliorative trends toward baseline. Furthermore, normal diet and Mediterranean diet interventions shifted the overall gut microbiota structure in MASLD mice similar to that of the NC group (Figure 8C).

At the genus level (Figures 8B, D), HF group mice exhibited significantly increased relative abundances of *Alistipes, Helicobacter, Mucispirillum*, and *Chlamydia* compared to the NC group (*p* < 0.05), whereas the abundances of *Prevotella, unclassified-f-Lachnospiraceae, Oscillibacter*, and *Roseburia* were markedly reduced (*p* < 0.05). However, in both HF-NC and HF-MD groups, the relative abundance of *Prevotella* significantly increased compared to the HF group, while *Helicobacter, Mucispirillum*, and *Chlamydia* showed pronounced decreases. There were no statistically significant differences found in these taxa between the HF-NC and HF-MD groups.

In summary, high-fat diet-induced MASLD mice exhibited diminished species richness of beneficial gut microbiota alongside elevated proliferation of pathogenic taxa. Mediterranean dietary intervention ameliorated gut microbial dysbiosis, demonstrating partial restoration of microbial community structure and conferring beneficial effects on intestinal homeostasis and metabolic health.

**Figure 9:**
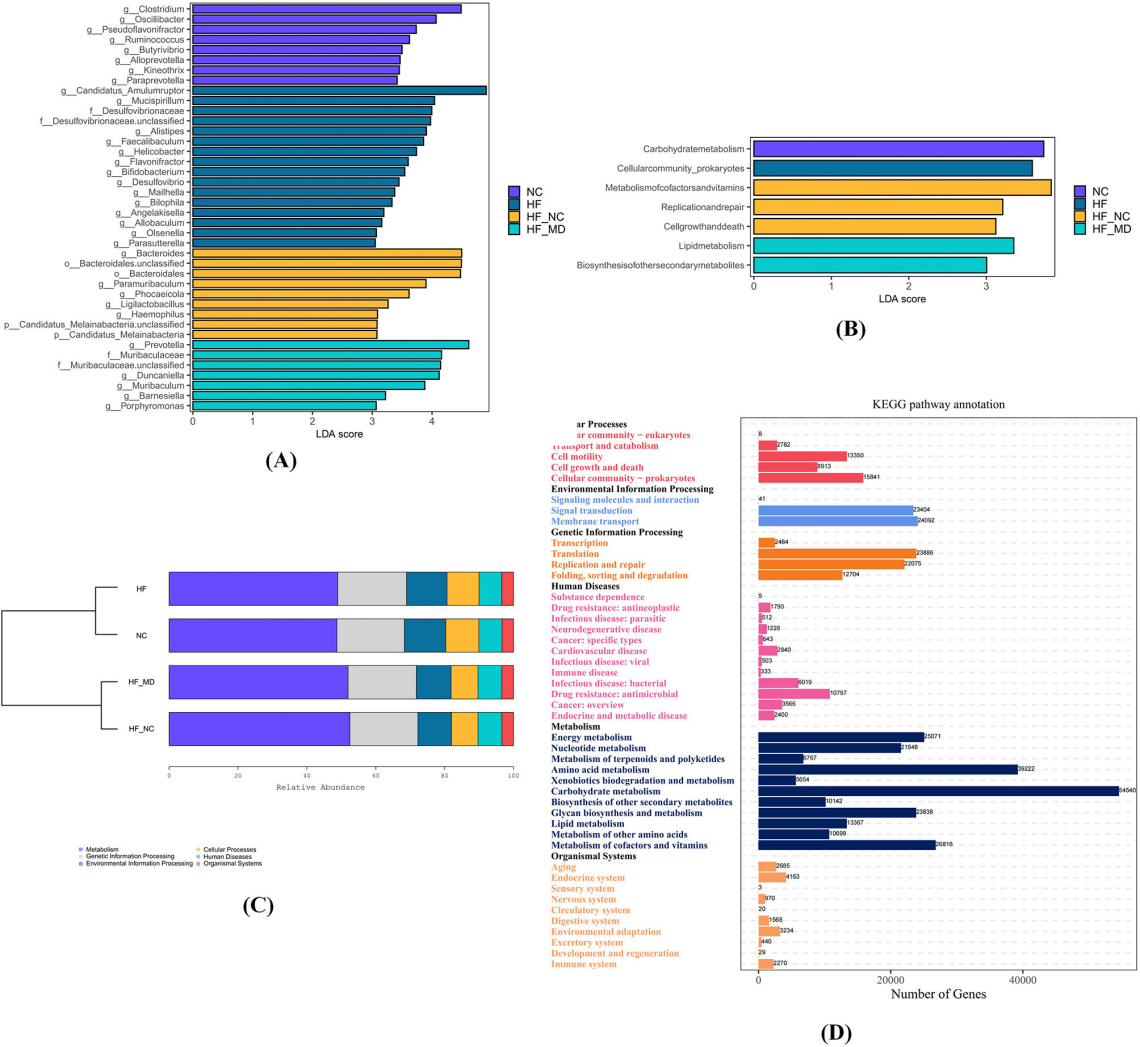
The four experimental groups exhibited distinct biomarker taxa with significant differential abundance. **(A)** LEfSe comparison of gut microbiota among the NC, HF, HF-NC, and HF-MD groups, filtered by an LDA score >3. **(B)** LEfSe comparison of differentially enriched KEGG pathways in gut microbiota among the NC, HF, HF-NC, and HF-MD groups, filtered by an LDA score >3. **(C)** KEGG functional analysis of gut microbiota demonstrated enrichment in metabolic pathways across all group. **(D)** Predominantly enriched metabolic pathways associated with gut microbial communities.

To identify statistically discriminative biomarker taxa across experimental groups and delineate key microbial responders to dietary regimens, LEfSe-generated cladograms were employed for phylogenetic feature selection. The analysis identified 8, 16, 9, and 7 differentially enriched taxa in NC, HF, HF-NC, and HF-MD groups, respectively (all LDA scores (log10) > 3) (Figure 9A). The NC group exhibited significant enrichment of commensal taxa including *Clostridium, Oscillibacter, Pseudoflavonifractor, Ruminococcus, Butyrivibrio, Alloprevotella*, and *Paraprevotella*. Conversely, HF group displayed elevated abundance of pathobionts such as *Faecalibaculum, Helicobacter, Flavonifractor, Desulfovibrio, Bilophila*, and *Parasutterella*. Dietary interventions modulated microbial profiles: HF-NC group demonstrated proliferation of beneficial genera (*Bacteroides, Paramuribaculum, Phocaeicola, Ligilactobacillus*), while HF-MD group showed dual enrichment patterns with beneficial taxa (*Prevotella, Muribaculum, Duncaniella, Barnesiella*) co-occurring with the opportunistic pathogen *Porphyromonas*.

To assess differences in gut microbial metabolic pathways between MASLD and NC mice, as well as alterations following dietary interventions, KEGG functional enrichment analysis was performed. Results revealed predominant clustering of core functionalities within central metabolic processes (Figure 9C). LEfSe analysis identified group-specific pathway enrichments: carbohydrate metabolism (NC group), prokaryotic cellular community organization (HF group), cofactor/vitamin metabolism, DNA replication/repair, and cell cycle regulation (HF-NC group), lipid metabolism and secondary metabolite biosynthesis (HF-MD group) (all LDA scores (log10) > 3) (Figure 9B).

KEGG pathway analysis revealed significant enrichment of gut microbiota in the following metabolic pathways (Figure 9D): Carbohydrate Metabolism, Amino Acid Metabolism, Metabolism of Cofactors and Vitamins, Energy Metabolism, Glycan Biosynthesis and Metabolism, Nucleotide Metabolism, Lipid Metabolism, Metabolism of Other Amino Acids, Biosynthesis of Other Secondary Metabolites, Metabolism of Terpenoids and Polyketides, and Xenobiotics Biodegradation and Metabolism.

#### 3.2.7 Quantification of microbial-derived short-chain fatty acids in the gut lumen

**Figure 10:**
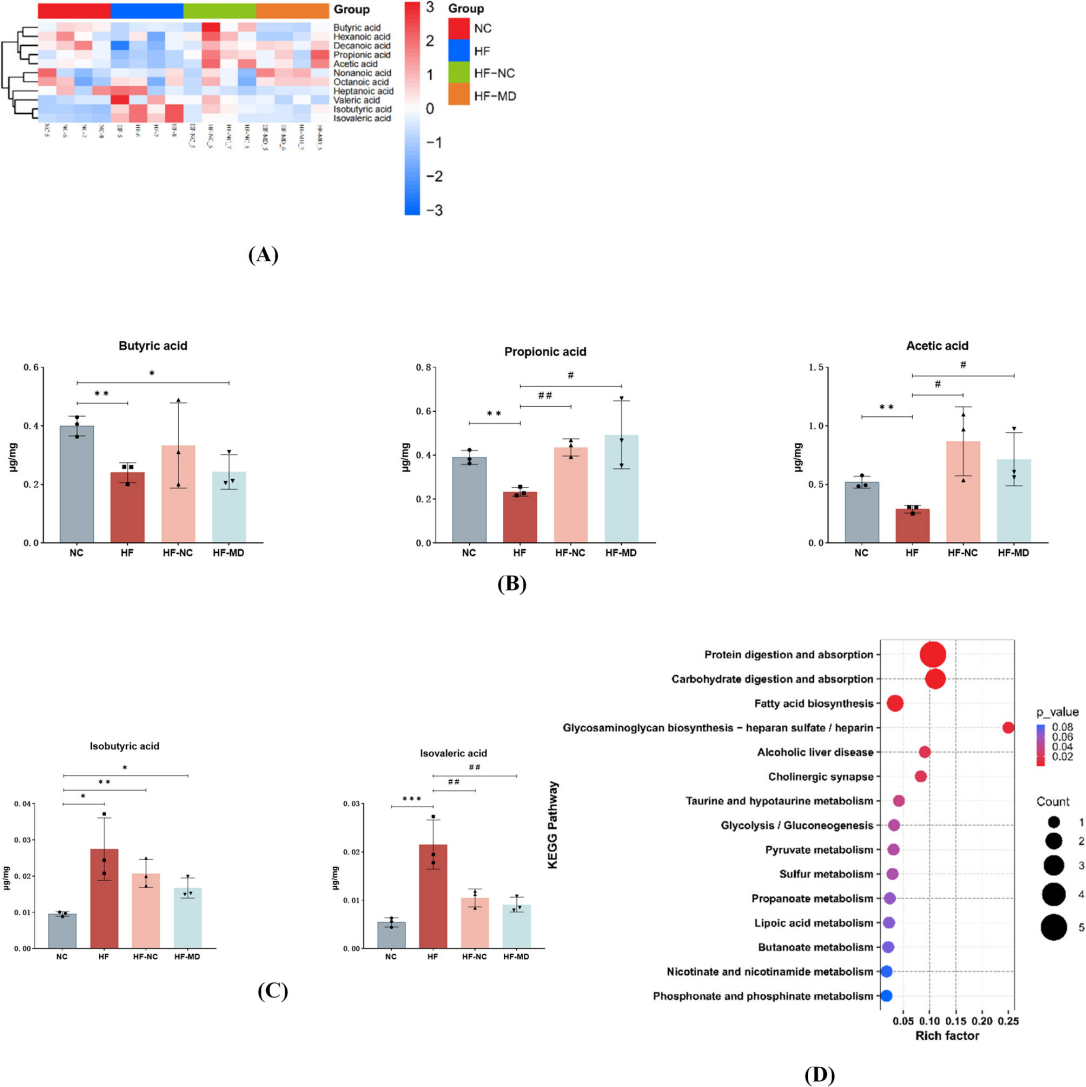
MASLD mice exhibited reduced production of SCFAs and increased BCFAs in the gut. MD intervention significantly elevated propionate levels while reducing isobutyric and isovaleric acids concentrations. **(A)** Heatmap analysis of 11 SCFAs in the gut microbiota. Color gradients represent Z-score normalized abundance values (scale: +3 to −3), with warm hues indicating higher relative abundance. **(B)** Comparative analysis of acetate, propionate, and butyrate levels across four groups. **(C)** Comparative analysis of isovalerate and isobutyrate levels among four groups. **(D)** KEGG functional enrichment of metabolic pathways associated with differentially abundant SCFAs. Significance thresholds were defined as follows: *p < 0.05, **p < 0.01, ***p < 0.001 vs. NC group; ^#^*p* < 0.05, ^##^*p* < 0.01 vs. HF group. (Only significant differences are marked).

The results demonstrated (as shown in Figure 10 and Table 1) that among the 11 quantified short-chain fatty acids (SCFAs), acetic acid constituted the predominant fraction, followed by propionic and butyric acids. The concentrations of total SCFAs in feces were significantly lower in the HF group when compared to the NC group (*p* < 0.05) (Figure 10A).

**Table 1:** Comparative analysis of acetic acid, propionic acid, butyric acid, and total SCFAs levels across experimental groups (μg/mg, mean ± SD).

**SCFAs**	**NC**	**HF**	**HF-NC**	**HF-MD**
Acetic acid	0.497 ± 0.061	0.271 ± 0.045	0.744 ± 0.346	0.622 ± 0.263
Propionic acid	0.371 ± 0.046	0.244 ± 0.026	0.480 ± 0.094	0.438 ± 0.167
Butyric acid	0.372 ± 0.062	0.247 ± 0.030	0.462 ± 0.283	0.228 ± 0.057
Total SCFAs	1.278 ± 0.1404	0.861 ± 0.082	1.750 ± 0.7104	1.340 ± 0.4831

Comparative analysis of gut microbial metabolites revealed distinct SCFA modulation patterns: the HF group showed depressed synthesis of primary SCFAs (acetic, propionic, and butyric acids) relative to NC controls (*p* < 0.05), contrasting with elevated branched-chain SCFAs (isobutyric/isovaleric acids; *p* < 0.05). Post-intervention groups (HF-NC/HF-MD) displayed partial normalization of SCFA composition toward NC-like profiles, though interventional strategies exhibited comparable efficacy without reaching statistical distinction (*p* > 0.05), likely reflecting power limitations inherent to the experimental design. The MD intervention demonstrated more pronounced propionate elevation coupled with greater attenuation of isobutyrate and isovalerate levels compared to conventional dietary regimens (Figures 10B, C).

The KEGG database was utilized to conduct pathway enrichment analysis on the differential metabolites, identifying significant enrichment in the following metabolic pathways: Protein digestion and absorption, carbohydrate digestion and absorption, Fatty acid biosynthesis, Glycosaminoglycan biosynthesis (heparan sulfate/heparin), Alcoholic liver disease, Cholinergic synapse, Taurine and hypotaurine metabolism, Glycolysis/gluconeogenesis, and Pyruvate metabolism (Figure 10D).

#### 3.2.8 Correlational analysis of gut microbiota, short-chain fatty acids, and MASLD-associated parameters

To identify pivotal gut microbial taxa and short-chain fatty acids (SCFAs) implicated in MASLD pathogenesis, Spearman correlation analysis was performed to evaluate associations among genus-level differential microbiota, SCFA profiles, and MASLD-related metabolic indices.

**Figure 11:**
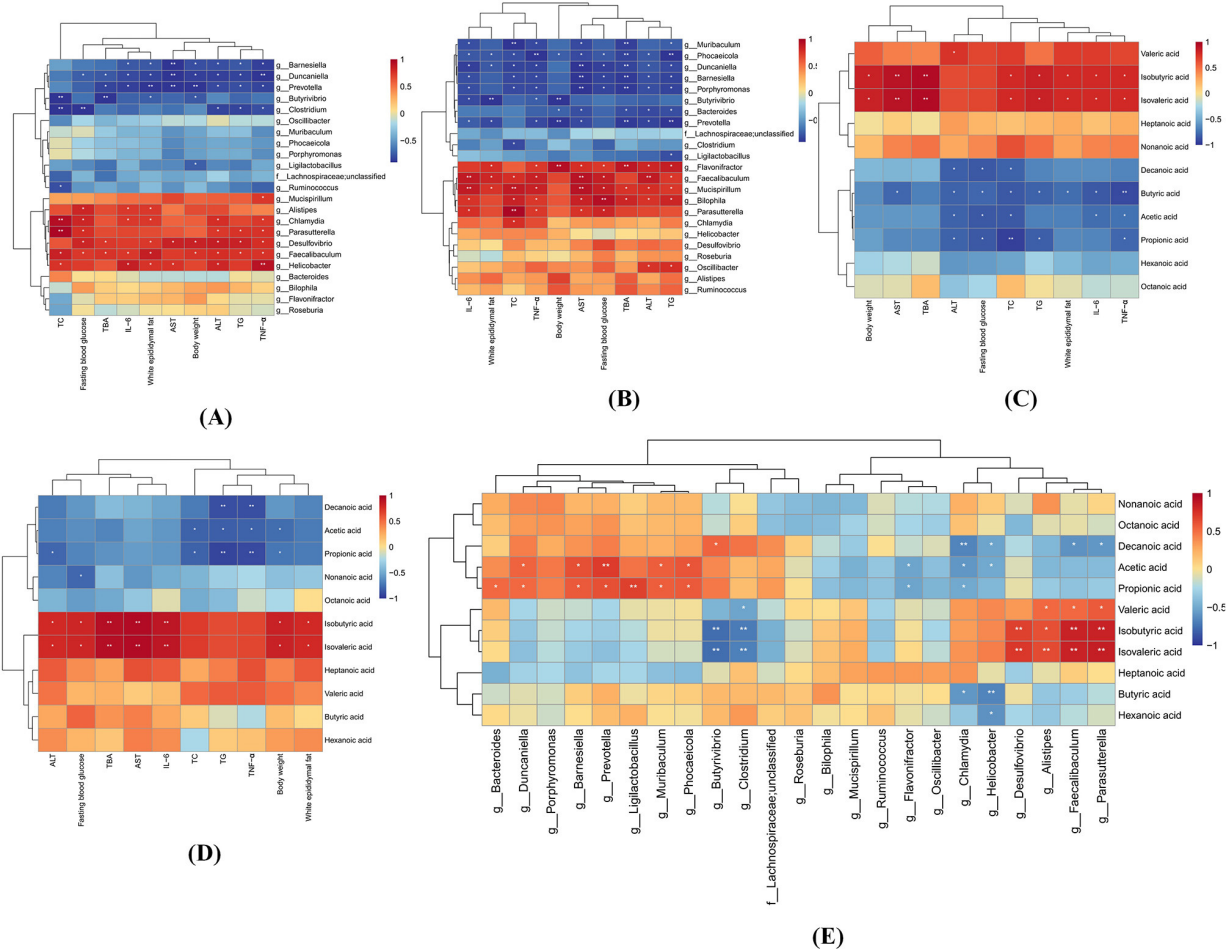
Stratified Spearman correlation analyses among gut microbiota, SCFAs, and MASLD-associated parameters at the genus level. **(A)** Heatmap depicting correlations between differential gut microbiota (HF vs. NC groups) and MASLD-related parameters. **(B)** Correlation heatmap of HF-MD vs. HF group differential microbiota with MASLD indicators. **(C)** Association profile of altered SCFAs (NC vs. HF groups) with MASLD biomarkers. **(D)** SCFA variation correlations between HF-MD and HF groups against MASLD parameters. **(E)** Microbial-SCFA interaction network across all experimental cohorts. Color gradients (red: positive; blue: negative) reflect correlation strength magnitudes. Asterisks denote statistical significance (*p < 0.05, **p < 0.01).

As depicted in Figures 11A, B, the color gradient (red to blue) reflects decreasing correlation strength. *Faecalibaculum, Desulfovibrio, Parasutterella*, and *Helicobacter* showed significant positive correlations with fasting blood glucose, TBA, serum TC, TG, ALT, and hepatic inflammatory cytokines (IL-6, TNF-α) (*p* < 0.05), consistent with their specific enrichment in the HF group, thereby supporting their pathogenic roles in MASLD progression. Conversely, *Barnesiella, Duncaniella*, and *Prevotella* exhibited marked inverse correlations with these metabolic and inflammatory markers (*p* < 0.05). Notably, *Faecalibaculum* and *Desulfovibrio* were positively associated with body weight, while *Prevotella, Duncaniella, Barnesiella*, and *Butyrivibrio* demonstrated negative weight correlations (*p* < 0.05), patterns replicated in both HF and HF-MD groups. The specific enrichment of *Prevotella, Duncaniella*, and *Barnesiella* in the HF-MD group further implicates these taxa as potential mediators of the weight-regulatory effects underlying MD intervention in MASLD.

The correlation analysis revealed distinct metabolic roles of SCFAs in MASLD progression (Figures 11C, D). BCFAs (isobutyric acid and isovaleric acid) showed significant positive associations (*p* < 0.05) with adverse metabolic parameters, including body weight, eWAT mass, TBA, serum TC, TG, AST, and hepatic IL-6, TNF-α, suggesting their potential as pathological promoters in MASLD. In stark contrast, straight-chain SCFAs (butyric acid, acetic acid, and propionic acid) exhibited protective effects through negative correlations (*p* < 0.05) with fasting blood glucose, serum TC, ALT, and hepatic TNF-α levels. Notably, intergroup comparative analysis revealed that propionic acid and acetic acid maintained consistent inverse correlations (*p* < 0.05) with serum TC, TG, hepatic TNF-α levels, and body weight across both HF and HF-MD cohorts. These robust associations, coupled with MD intervention-specific alterations in propionate profiles, strongly suggest that the therapeutic efficacy of MD against MASLD may be mechanistically rooted in its capacity to modulate these key SCFAs, thereby attenuating lipid dysmetabolism and inflammation central to disease progression.

The analysis of the relationship between gut microbiota and SCFAs revealed the following associations (Figure 11E): Isobutyric acid and Isovaleric acid exhibited significant positive correlations with *Faecalibaculum, Parasutterella, Alistipes*, and *Desulfovibrio* (*p* < 0.05), while demonstrating notable negative associations with *Butyrivibrio* and *Clostridium* (*p* < 0.05). Similarly, Propionic acid and Acetic acid showed significant positive correlations with *Duncaniella, Barnesiella, Prevotella*, and *Muribaculum* (*p* < 0.05), whereas significant negative correlations were observed with *Flavonifractor* and *Chlamydia* (*p* < 0.05).

Collectively, these findings suggest that Isobutyric acid, Isovaleric acid, *Faecalibaculum, Parasutterella*, and *Desulfovibrio* exert detrimental effects during the pathogenesis of MASLD. Conversely, Propionic acid, Acetic acid, *Duncaniella, Barnesiella*, and *Prevotella* demonstrate significant protective roles in ameliorating MASLD, potentially serving as key mediators through which MD intervention mitigates MASLD progression. Consequently, MD remodels the gut microbial ecosystem and metabolites (notably propionate/acetate production) to modulate host metabolism, highlighting therapeutic targets for MASLD.

## 4 Discussion

MASLD is characterized by hepatic fat accumulation exceeding 5% in conjunction with at least one metabolic abnormality (e.g., obesity, hypertension, dyslipidemia, or insulin resistance). Its pathogenesis is closely linked to energy surplus, sedentary behavior, and genetic predisposition, representing the hepatic manifestation of metabolic syndrome. Although often asymptomatic in early stages, MASLD may progress to steatohepatitis (MASH), fibrosis, cirrhosis, or hepatocellular carcinoma over time. Current understanding of MASLD mechanisms has transitioned from the conventional “two-hit theory” to a “multiple parallel hits” framework, emphasizing the synergistic activation of metabolic dysregulation, oxidative stress, inflammatory responses, and gut microbiota imbalance in driving disease progression. The recent nomenclature update from NAFLD to MASLD reflects the need to better delineate its core metabolic associations, eliminate ambiguities in the “non-alcoholic” terminology, and underscore the importance of multidisciplinary management for preventing hepatic and extrahepatic complications. The primary approach to treatment focuses on changes in lifestyle, such as losing weight, improving diet, and engaging in consistent physical activity.

Dietary intervention remains foundational in MASLD management, though consensus on optimal nutritional strategies remains elusive. MD emphasizing plant-based foods such as whole grains, fruits, vegetables, legumes and nuts, along with moderate consumption of dairy and fish, while limiting red meat and processed items, shows a variety of advantages. Meta-analyses indicate MD improves metabolic parameters through weight loss, anti-inflammatory/antioxidant effects, and gut microbiota modulation (17). Compared to low-fat diets (LFDs), MD exhibits superior efficacy in reducing ALT, TG, TG/HDL-C ratios, and fatty liver index (FLI) (18), while outperforming Dietary Approaches to Stop Hypertension (DASH) in systolic blood pressure reduction (19). Low-carbohydrate diets (LCDs), particularly ketogenic regimens, show short-term hepatic fat reduction (20) but pose risks of dyslipidemia, gastrointestinal disturbances, and nephrolithiasis due to nutrient imbalances. Intermittent fasting (IF) may confer hepatic benefits (21); however, some studies associate it with elevated atherosclerosis and cardiovascular mortality risks (22, 23). Vegetarian diets improve MASLD via lipid metabolism regulation and gut microbiome modulation (24), though deficiencies in vitamin B12, iron, and ω-3 polyunsaturated fatty acids (PUFAs) require monitoring (25). While DASH demonstrates potential metabolic benefits, its restrictive sodium limitations impair adherence, and MASLD-specific evidence remains inconclusive (26). Current evidence indicates that the MD demonstrates comprehensive metabolic regulatory benefits through the synergistic effects of substituting saturated fatty acids (SFAs) with monounsaturated fatty acids (MUFAs) and incorporating high-fiber complex carbohydrates. However, the specific therapeutic effects and underlying mechanisms by which MD ameliorates MASLD remain to be fully elucidated. This study aims to provide more systematic scientific evidence.

### 4.1 Hepatic metabolic regulation and inflammatory modulation

The high-fat diet (HF) is well-established for inducing MASLD in experimental animals (27). Consistent with previous findings (28), an HF-induced MASLD mouse model was successfully established in this study, recapitulating key pathological features including marked body weight gain, increased visceral adiposity, dysregulated glucose and lipid metabolism, hepatic steatosis, and inflammatory infiltration (Figure 2). These phenotypic alterations were accompanied by significant dysregulation of gut microbiota composition and microbial metabolites, characterized by reduced α-diversity, disrupted community structure, and decreased fecal SCFAs production. Notably, intervention with either normal chow or MD reversed hepatic steatosis, attenuated hepatic inflammation, and partially restored gut microbial homeostasis, with MD demonstrating superior efficacy in body weight reduction and metabolic improvement compared to normal chow.

Our findings align with meta-analytical evidence (29), which indicates that ≥5% body weight loss improves hepatic and metabolic parameters, while ≥7% loss significantly ameliorates liver histology and function. Pronounced weight reduction was observed in both dietary intervention groups, with MD achieving a more substantial decrease (>12%), underscoring the pivotal role of dietary patterns in modulating obesity-related metabolic dysregulation. Previous studies further highlight MD's potential to reduce central obesity and visceral adiposity (30, 31). In this study, dietary interventions reduced body weight and showed a downward trend in liver index, though intergroup differences in hepatic index improvement were not statistically significant. By contrast, eWAT mass was markedly reduced, particularly following MD intervention. This suggests adipose tissue may respond more rapidly to MD, whereas significant amelioration of visceral steatosis may require prolonged intervention.

Our results demonstrate that MD significantly reduced serum TG and TC levels compared to normal chow intervention (Figure 4), consistent with the meta-analytical evidence reported by Omid Asbaghi et al. (32). This metabolic advantage of MD further underscores its superior lipid-modulating properties, likely attributable to its unique nutritional profile enriched with monounsaturated/polyunsaturated fatty acids (MUFAs/PUFAs), polyphenols, and dietary fiber. Specifically, ω-3 PUFAs may mediate lipid homeostasis through PPARα activation, enhancing fatty acid oxidation, suppressing lipogenesis, promoting bile acid secretion and cholesterol excretion, and improving adipocyte function via adiponectin (33). Furthermore, polyphenolic compounds such as hydroxytyrosol (HT) and oleuropein (OLE) exhibit dual protective effects by preserving HDL from oxidative damage and enhancing cholesterol clearance through AMPK activation-mediated upregulation of LDL receptor expression, thereby optimizing lipid metabolism (34).

The bioactive components in MD demonstrate synergistic anti-inflammatory and antioxidant properties. Our analysis revealed markedly elevated serum IL-6 and TNF-α levels in MASLD mice, which were effectively suppressed by MD intervention, showing superior anti-inflammatory efficacy compared to normal chow (Figure 4). Recent investigations demonstrate that HT from olive mill wastewater (MD) suppresses IL-6 gene transcription (35), while OLE interrupts LPS-induced TLR4-MyD88-NF-κB/MAPK signaling (36), forming a dual anti-inflammatory barrier. Mitochondrial dysfunction in MASLD progression involves ROS-mediated membrane destabilization, electron transport chain impairment, and lipid peroxidation, forming a self-perpetuating pathogenic cycle (37). Mechanistically, HT enhances Nrf2/ARE-driven antioxidant enzymes (SOD, catalase), whereas resveratrol and quercetin cooperatively suppress NADPH oxidase activity (38, 39), collectively disrupting the “oxidative stress—mitochondrial damage—inflammatory amplification—secondary oxidative stress” axis to prevent steatosis-to-MASH transition. Although MD exhibited greater ALT/AST reduction than ND, clinical comparisons of dietary regimens remain inconclusive (40). Marin-Alejandre et al.'s RCT (41) reported equivalent ALT/γ-GT improvements with AHA diet and MD, yet divergent AST responses, suggesting intervention outcomes depend on metabolic heterogeneity and nutrient interactions.

### 4.2 Gut microbiota remodeling

The gut-liver axis (GLA), a bidirectional signaling network connecting intestinal and hepatic systems, critically regulates liver-gut homeostasis through microbial metabolism, immune signaling, and metabolite exchange. In MASLD pathogenesis, GLA dysfunction exhibits a dual-hit pathology: compromised intestinal barrier integrity synergizes with gut dysbiosis to drive disease progression (42). For instance, gut dysbiosis-induced barrier disruption facilitates translocation of microbial lipopolysaccharide (LPS) into circulation, triggering IL-6/TNF-α release and NADPH oxidase-mediated oxidative stress. This cascade not only directly induces hepatocyte injury but also activates hepatic stellate cells (HSCs) to accelerate fibrogenesis (10, 43, 44).

While current evidence remains fragmented regarding microbial dynamics in MASLD progression and dietary interventions, emerging studies consistently identify disease-associated dysbiotic patterns. Clinical observations reveal characteristic microbial shifts in MASLD patients: marked elevation of potential pathogens (Enterobacteriaceae, Escherichia) alongside depletion of metabolically beneficial commensals (Bacteroides, Bifidobacterium), with exacerbation during fibrotic progression (10). Systematic microbiota profiling revealed characteristic HF-induced restructuring in MASLD (Figures 7, 8). HF triggered phylum-level dysbiosis, marked by increased *Proteobacteria*/*Actinobacteria* and reduced *Firmicutes* abundance. Notably, genus-level analysis demonstrated contrasting *Prevotella* depletion and *Alistipes* enrichment, aligning with clinical observations of progressive *Firmicutes* reduction and early *Prevotella* decline in MASLD (45). While *Firmicutes* [often termed “obesity-associated bacteria” for their metabolic regulatory roles (46)] typically expand under HF, their suppression here may reflect chronic HF-induced intestinal inflammation that selectively inhibits oxidative stress-sensitive genera (e.g., *Lactobacillus*) while promoting inflammation-resistant *Proteobacteria* (e.g., Enterobacteriaceae). Intriguingly, *Alistipes* enrichment in this MASLD model contrasts with its reported depletion in cirrhosis studies (47, 48), despite proposed anti-inflammatory properties (49, 50). These findings suggest that MASLD mouse models partially recapitulate human gut microbial communities, although notable inter-species differences persist. Furthermore, microbial functionality exhibits significant context-dependence, with biological effects likely collectively modulated by host dietary components, local metabolites, and microbial interaction networks.

Both normal chow and Mediterranean diet (MD) interventions ameliorated gut dysbiosis in MASLD mice, characterized by increased abundance of *Prevotella* and *Lachnospiraceae*. While ND primarily reduced opportunistic pathogens, MD specifically enriched beneficial genera (*Prevotella, Muribaculum, Duncaniella, Barnesiella*), whose levels positively correlated with propionate production. The therapeutic efficacy of MD arises from multicomponent synergy: ω-3 PUFAs modulate the *Firmicutes/Bacteroidetes* ratio, enhancing *Lachnospiraceae* and *Bifidobacteriaceae* while suppressing LPS-producing *Enterobacteriaceae* (51); dietary fibers foster SCFA-producing bacteria through diversified fermentation substrates (52); and polyphenols selectively promote *Lactobacillus* growth, inhibit *Enterococcus*, and upregulate intestinal tight junction proteins (53). Notably, *Prevotella* may mediate MD's metabolic effects via carbon/BCAA-related fatty acid pathways (54), though conflicting clinical evidence links *P. copri* abundance to MASLD progression in Asian populations, suggesting strain-specific functionality (55). Importantly, not all studies support MD's microbial modulation—a 6-month trial (56) observed comparable gut microbiota alterations between MD and Western diets, highlighting challenges in achieving significant short-term restructuring of stable microbial ecosystems.

The downstream effects of gut-derived metabolites, particularly SCFAs, may constitute a pivotal mechanistic pathway. Beyond serving as primary energy substrates for colonocytes, SCFAs enhance intestinal barrier integrity by reducing systemic LPS levels (57). Their deficiency exacerbates hepatic inflammation and insulin resistance through impaired anti-inflammatory responses and attenuated HDAC3 activity (10, 44). Our targeted metabolomics revealed marked SCFA dysregulation in MASLD mice: decreased metabolically protective acetate, propionate, and butyrate alongside elevated detrimental isobutyrate/isovalerate (Figures 10, 11). MD intervention partially reversed this profile, promoting propionate accumulation while suppressing branched-chain fatty acids (BCFAs). MD's abundant dietary fibers reshape SCFAs synthesis by providing diverse fermentation substrates. Elevated propionate activates the APN-AMPK-PPARα axis, suppressing *de novo* lipogenesis while enhancing β-oxidation to alleviate hepatic lipid accumulation (58). Concurrent butyrate upregulation may mitigate mitochondrial dysfunction and ROS production through phage-bacterial LPS interactions, thereby reducing hepatocyte injury (59).

BCFAs, including isobutyrate and isovalerate, are microbial proteolytic metabolites derived from branched-chain amino acid (BCAA) fermentation, exhibiting detrimental effects on intestinal and metabolic homeostasis (60, 61). In the distal colon under high-fat diet conditions—characterized by limited fermentable carbohydrates—gut microbiota preferentially engage in proteolytic metabolism, driving BCFAs overproduction (62). This metabolic shift correlates with hepatic pathophysiology: murine models of macrovesicular steatosis demonstrate elevated cecal BCFAs levels concomitant with insulin resistance and hyperleptinemia (63), while microbial-derived phenylacetic acid exacerbates hepatic lipid accumulation via TCA cycle-potentiated BCAA catabolis (61). Collectively, these findings position proteolytic metabolites as critical mediators of steatosis progression. Dietary strategies favoring carbohydrate over proteolytic fermentation, such as increased fiber intake, may mitigate metabolic disorders including MASLD. The Mediterranean diet (MD) exemplifies this approach by supplying abundant fermentable fiber to suppress proteolytic pathway dominance and inhibit proteolytic taxa proliferation, as evidenced in our study. However, our study lacks comprehensive analysis of intestinal protein fermentation metabolites, including indole derivatives and branched-chain fatty acids. The mechanisms by which these metabolites influence MASLD pathogenesis remain unclear. Future research should investigate their roles in MASLD progression and evaluate dietary interventions targeting protein quality, intake levels, and fermentability to optimize clinical management.

## 5 Conclusions

This study systematically delineates the multidimensional therapeutic mechanisms of MD in ameliorating high-fat diet-induced MASLD through metabolic-inflammatory-gut microbiota crosstalk. MD demonstrated superior efficacy over normal chow by significantly reducing body weight, improving glucolipid metabolism, reversing hepatic steatosis/inflammatory infiltration, and restoring gut microbial homeostasis through selective enrichment of beneficial taxa (*Prevotella, Muribaculum*) and metabolic reprogramming (enhanced protective SCFAs vs. suppressed detrimental BCFAs), thereby establishing a coordinated microbiota-metabolite-host interaction network. Nevertheless, limitations remain. Subsequent studies should combine hepatic transcriptomic-metabolomic profiling in murine models to deeply resolve MD's regulatory nodes in liver metabolism and liver-gut feedback pathways, while further investigating functional inconsistencies in specific microbiota (e.g., *Alistipes*) and mechanisms of proteolytic metabolites. Long-term clinical follow-up models are also warranted to evaluate MD's preventive value against MASLD, cirrhosis, and extrahepatic complications.

## Data Availability

The original contributions presented in the study are publicly available. This data can be found in here: https://www.ncbi.nlm.nih.gov/, accession number PRJNA1297975.
